# An ERP Assessment of Hemispheric Projections in Foveal and Extrafoveal Word Recognition

**DOI:** 10.1371/journal.pone.0023957

**Published:** 2011-09-15

**Authors:** Timothy R. Jordan, Giorgio Fuggetta, Kevin B. Paterson, Stoyan Kurtev, Mengyun Xu

**Affiliations:** Faculty of Medicine and Biological Sciences, University of Leicester, Leicester, United Kingdom; Washington University School of Medicine, United States of America

## Abstract

**Background:**

The existence and function of unilateral hemispheric projections within foveal vision may substantially affect foveal word recognition. The purpose of this research was to reveal these projections and determine their functionality.

**Methodology:**

Single words (and pseudowords) were presented to the left or right of fixation, entirely within either foveal or extrafoveal vision. To maximize the likelihood of unilateral projections for foveal displays, stimuli in foveal vision were presented away from the midline. The processing of stimuli in each location was assessed by combining behavioural measures (reaction times, accuracy) with on-line monitoring of hemispheric activity using event-related potentials recorded over each hemisphere, and carefully-controlled presentation procedures using an eye-tracker linked to a fixation-contingent display.

**Principal Findings:**

Event-related potentials 100–150 ms and 150–200 ms after stimulus onset indicated that stimuli in extrafoveal and foveal locations were projected unilaterally to the hemisphere contralateral to the presentation hemifield with no concurrent projection to the ipsilateral hemisphere. These effects were similar for words and pseudowords, suggesting this early division occurred before word recognition. Indeed, event-related potentials revealed differences between words and pseudowords 300–350 ms after stimulus onset, for foveal and extrafoveal locations, indicating that word recognition had now occurred. However, these later event-related potentials also revealed that the hemispheric division observed previously was no longer present for foveal locations but remained for extrafoveal locations. These findings closely matched the behavioural finding that foveal locations produced similar performance each side of fixation but extrafoveal locations produced left-right asymmetries.

**Conclusions:**

These findings indicate that an initial division in unilateral hemispheric projections occurs in foveal vision away from the midline but is not apparent, or functional, when foveal word recognition actually occurs. In contrast, the division in unilateral hemispheric projections that occurs in extrafoveal locations is still apparent, and is functional, when extrafoveal word recognition takes place.

## Introduction

For many years (e.g., [1]), research using lateralised visual displays has shown that words presented outside the fovea are processed more efficiently in the right visual hemifield (RVF) than in the left visual hemifield (LVF; for overviews, see [2], [3]). There seems little doubt that this difference between RVF and LVF presentations reflects the existence and influence of unilateral contralateral projections to the left hemisphere (LH) and right hemisphere (RH), respectively. However, while the unilateral projection of words to each contralateral hemisphere is well-established for locations outside the fovea, the projection of words in foveal locations is a matter of debate in visual word recognition research.

One view is that each fovea is divided precisely at its vertical midline so that all information either side of this midline projects unilaterally to the contralateral hemisphere, with the result that hemispheric asymmetries affect word recognition right up to the point of fixation (for reviews, see [3]–[5]). Thus, according to this “split fovea theory” of word recognition (hereafter SFT), each fovea contains only unilateral projections to each contralateral hemisphere, and the division between these contralateral projections at the vertical midline is so precise that it occurs with no amount of bilateral overlap between the projection of information from the left and right hemifields.

However, many have argued that although unilateral contralateral projections from the left and right hemifields are likely to exist in foveal vision, these projections are unlikely to be divided precisely at the vertical midline (for reviews, see [2], [3]). Instead, a considerable body of evidence indicates a region in foveal vision around the vertical midline in which an intermingling of ganglion cells projects contralaterally and ipsilaterally such that information falling in this region projects to both hemispheres (for relevant reviews, findings, and opinions, see [2], [3], [5]–[20]; see also Footnote S1). However, the size of this region of bilateral projection has yet to be established (e.g., [21]–[23]) and is unlikely to extend across the entire fovea, with the effect that foveal projections become increasingly contralateral away from the midline and provide increasingly divided unilateral inputs to each contralateral hemisphere. Indeed, divided unilateral contralateral projections to the LH and RH may become predominant in foveal vision away from the midline, as well as outside the fovea.

However, despite the likelihood of an anatomical division in unilateral contralateral projections within the fovea away from the midline, numerous studies have shown no indication that such a division is functional for word recognition when care has been taken to position stimuli accurately within foveal vision. For example, Jordan, Paterson and Stachurski [24] used fixation-contingent displays to present words in precisely matched locations each side of fixation, either entirely within foveal or extrafoveal vision. Jordan et al. found a strong recognition advantage for words presented to the right of fixation in extrafoveal vision but no advantage for the same words presented to the right of fixation in foveal vision, despite the precaution that words in foveal vision were presented slightly away from fixation to avoid areas of bilateral projection, and extended towards the edges of the fovea where unilateral contralateral projections were most likely to exist. Consequently, these findings are consistent with a functional division in hemispheric projections for words encountered outside foveal vision but indicate no functional division for words within foveal vision (see also [25]). Other studies have provided similar evidence, using a variety of paradigms and procedures (see [3], [11] for reviews).

However, as Jordan and Paterson [3], [11] point out, this lack of evidence for a division in unilateral contralateral foveal projections that is functional for foveal word recognition need not mean that an anatomical division does not exist in foveal vision. Indeed, although SFT's proposal of a precise anatomical division at the vertical meridian appears implausible, an anatomical division in unilateral contralateral projections in the fovea away from midline areas of bilateral projection is more likely to exist. If such a division in foveal vision could be revealed, a concurrent investigation of its influence on word recognition would provide a major advance in determining whether divided unilateral contralateral foveal projections have functional relevance for foveal word recognition.

The purpose of the present study, therefore, was to investigate the existence of a division in unilateral contralateral projections in foveal vision away from the midline and, if found, to establish whether this division produces concomitant influences on foveal word recognition. An effective method for investigating these interrelated issues is to combine the precise placement of words in foveal vision, using an eye-tracker linked to a fixation-contingent display, with behavioural measures of word recognition (reaction times and accuracy) and on-line monitoring of the hemispheric activity evoked by stimuli presented in each hemifield using event-related potentials (ERPs). ERPs measure changes in electrical voltage above the scalp, evoked by electrical activity in the brain produced when participants process visually-presented stimuli [26]. In contrast to behavioural measures alone, which represent the combination of all processing stages from early visual processing to behavioural response (e.g., a key press), the ERP technique can provide fine-grained information about the time course of neural processing in each hemisphere with excellent temporal resolution [27], [28].

The potential of ERPs for revealing a division in unilateral contralateral projections in foveal vision is demonstrated by a study by Martin, Thierry, Démonet, Roberts, and Nazir [29] which focused on the P100 component of early perceptual analyses (e.g., [30]–[32]). Five-letter words and pseudowords were displayed briefly so that each stimulus straddled a central fixation point at various locations. When stimuli were presented almost entirely to either the left or right of the fixation point, the P100 peak was delayed in the hemisphere ipsilateral to the side of stimulus presentation. As Martin et al. suggest, this P100 delay may have reflected a division in hemispheric projections in foveal vision in which visual information from each side of fixation was projected unilaterally to the contralateral hemisphere and was ultimately transferred to the ipsilateral hemisphere via the splenium of the corpus callosum.

However, the stimuli used by Martin et al. [29] were unusually large (6.65^0^ in width) and extended a considerable distance outside the fovea into regions where unilateral projections to each contralateral hemisphere are already well established (see e.g., [1]–[3]). Moreover, although fixation accuracy was critical to ensure the correct retinal location of each stimulus, no external monitoring or control was used to determine where fixations actually occurred in this study [3], [33]–[35]. Consequently, it is not clear that the indication of a division in unilateral hemispheric projections reported by Martin et al. was due to projections within the fovea. Moreover, the time window of the P100 wave of around 100–150 ms post-stimulus-onset is an early stage at which to assess effects on word recognition. Consequently, even if a division in unilateral projections exists in foveal vision, transmission of visual information between the two hemispheres via callosal fibres may be so rapid that an initial division in hemispheric processing is not functionally relevant for later stages of processing when word recognition actually takes place (e.g., [36]). Indeed, the P100 latencies observed by Martin et al. were not affected by lexicality, suggesting that this early ERP component was not actually related to lexical processing. Nevertheless, it is clear that, by using appropriate experimental procedures and controls, combining ERP and behavioural measures of word recognition may offer important new insight into the role of divided hemispheric projections in foveal word recognition.

Accordingly, the present research investigated the existence of divided hemispheric projections in foveal vision and their functionality for word recognition by using a paradigm designed to obtain accurate ERP and behavioural evidence of hemispheric projections and word recognition at precisely-controlled retinal locations. Single words and pseudowords were presented unilaterally in each visual hemifield at eccentricities that placed them entirely in either foveal or extrafoveal vision. This provided 4 target locations: LVF Extrafovea, LVF Fovea, RVF Fovea, RVF Extrafovea. Presentations in extrafoveal locations were included because divided unilateral contralateral projections are well-established in these locations [1]–[3] and so these locations provided important benchmarks against which the ERP and behavioural findings obtained for foveal locations could be compared. To avoid midline areas of bilateral projection for foveal stimuli and to occupy foveal areas where unilateral contralateral projections are most likely to exist, the nasal edges of stimuli in foveal vision were presented 0.10^0^ from fixation and stimuli extended to the temporal edges of foveal vision. The nasal edges of stimuli in extrafoveal vision were presented 2.00^0^ from fixation, following previous research showing clear evidence of divided unilateral contralateral projections at this eccentricity and beyond (e.g., [2], [3], [5], [24], [25]). An eye-tracking system linked to a computer-controlled, fixation-contingent display ensured accurate fixation when each stimulus was presented, and ensured that all stimuli were displayed at precisely the required retinal locations. A lexical decision task, which provides both reaction time and accuracy measures of performance, was used to provide behavioural measures of word recognition. The task is well-suited to this purpose because it requires lexical access and allows confounds present in other behavioural tasks to be avoided (see Footnote S2). To quantify the timing of the projection of stimuli to each hemisphere and the time course of word recognition, post-stimulus-onset latencies were measured in each hemisphere for three ERP peaks (P100, N170, and P325) that are prominent in the waveforms seen for words and pseudowords in the parietooccipital region and which provide good indications of the onset of processes ranging from early perceptual analysis to lexical selection (e.g., [37]–[39]). The P100 component peaks at around 100–150 ms post-stimulus-onset and reflects early perceptual processing (e.g., [29], [32], [40]). This is followed by the N170 which is a posterior negative component that peaks at around 150–200 ms and occurs for highly familiar stimuli, such as words, and appears to reflect sub-lexical processing of letter identities and letter combinations (e.g., [39], [41]–[43]). The time course of these early components terminates with the P325, a posterior positive component that peaks at around 300–350 ms and corresponds to the selection of a single whole-word orthographic representation from a number of possible candidates that are compatible with incoming information (i.e., a high-level lexical selection process [37]).

Several key predictions were made. An anatomical division which causes stimuli in each visual hemifield to project unilaterally to the contralateral hemisphere should be revealed by the relative latency of the P100 in each hemisphere. In particular, the P100 should be observed earlier for the hemisphere contralateral (rather than ipsilateral) to the hemifield in which a stimulus is presented. This asymmetry should be clear for stimuli in extrafoveal locations, where the presence of unilateral contralateral projections is well-established, but if the anatomical division in hemispheric projections extends into foveal vision, a P100 asynchrony should also be observed for stimuli in foveal locations. If a division (foveal or extrafoveal) observed for the P100 is maintained at later stages in processing (N170, P325), the same hemispheric asynchrony should also be observed for these later components. But if an initial division in hemispheric projections is reduced or removed at later stages (N170, P325), the asynchronies observed across the two hemispheres for these later components should be smaller or completely removed. Finally, if an anatomical division (foveal or extrafoveal) in hemispheric projections is functional for word recognition, words should produce a behavioural advantage when presented in the RVF since an anatomical division would cause these words to project to the language-dominant LH. This asymmetry in performance should be clear for stimuli in extrafoveal locations. Crucially however, if an anatomical division in foveal vision is revealed by the ERP evidence but has no functionality for word recognition, words in foveal vision should produce similar levels of performance in each visual hemifield despite this division.

## Methods

### Ethics Statement

This research was conducted with the approval of the School of Psychology Research Ethics Committee at the University of Leicester, and in accordance with the ethical guidelines of the British Psychological Society. All participants understood the information given about electroencephalographic recording and gave written informed consent according to the Declaration of Helsinki.

### Participants

Twelve native English speakers, 18–32 years of age, took part in the experiment. All participants had at least normal or corrected to normal acuity, determined by a Bailey-Lovie eye chart, were right handed as assessed by a revised Annett Handedness Questionnaire [44], [45], and were right-eye dominant as determined using both the Miles test [46] and the Porta test [47]. All participants were selected to be LH-dominant for language and had previously shown the well-established LH advantage for words presented in extrafoveal locations.

### Stimuli

Stimuli were 115, 5-letter English words and 115, 5-letter pronounceable pseudowords generated from the words by substitution of one letter at any of the 5 possible locations (e.g., TABLE, TUBLE). Written frequency of word stimuli was between 100 and 300 per million (mean = 171 per million) according to the CELEX database [48]. Forty-eight additional stimuli (24 words and 24 pseudowords) were used as practice items at the start of each session. Stimuli were presented to the left and right of a central fixation point in either foveal or extrafoveal vision, in Courier new font. As described previously, the locations of foveal and extrafoveal stimuli were selected to maximise the likelihood of unilateral projections to each contralateral hemisphere. For foveal stimuli, this involved avoiding midline areas of bilateral projection and selecting the physical size of foveal stimuli to ensure they extended to the edges of foveal vision but not beyond. Accordingly, the nasal edges of foveal stimuli were 0.10^0^ from fixation and these stimuli subtended 1.25^0^ in width. The physical size of extrafoveal stimuli was adjusted to remove the substantial confounding differences in overall visibility between foveal and extrafoveal locations that would otherwise have occurred [49]. The nasal edges of extrafoveal stimuli were 2.00^0^ from fixation and these stimuli subtended 3.75^0^ in width.

### Apparatus

Stimuli were presented on a high-definition display, a Cambridge Research Systems VSG 2/5 card controlled stimulus presentations, and responses were collected via a Cambridge Research Systems CT3 response box. The experiment was conducted in a sound-attenuated and darkened room. Stimulus viewing was monocular via each participant's dominant eye to eliminate confounding effects of binocular fixation disparity [50], [51] and each non-dominant eye was occluded using a light-proof eye-patch (Cambridge Research Systems). The fixation location of each dominant eye was monitored using a Skalar IRIS eye-tracking system (Cambridge Research Systems) clamped to each participant's head, and this in turn was clamped in a head brace and chin rest throughout the experiment to prevent movement. This arrangement allowed the eye-tracking system to consistently measure and control fixation location to within 2′ of arc at a sampling rate of 1000 Hz, and so provided the precision required for ensuring the accurate presentation of foveal and extrafoveal stimuli throughout the experiment. The output of the tracker was recorded through the ADC input of the VSG2/5 card, which also controlled the visual display (for further details, see [52]).

### Design

Each participant took part in a single session which consisted of 48 practice items followed by 5 blocks of 184 stimuli, each separated by a 5-min rest. Within each block, equal numbers of words and pseudowords were selected pseudo-randomly and assigned pseudo-randomly to the four stimulus locations: LVF Extrafovea, LVF Fovea, RVF Fovea, RVF Extrafovea. Across all blocks, each participant was shown all 230 experimental stimuli once at each stimulus location.

### Procedure

At the start of each session, participants were given instructions describing the lexical decision task and emphasizing the importance of speed and accuracy when responding. The eye-tracking system was then calibrated. At the start of each trial, a small fixation point was presented at the centre of the screen. Participants were required to fixate this point and target presentation was prevented until accurate fixation occurred continuously for 300 ms. When this criterion was satisfied, a word or pseudoword was shown for 150 ms at one of the four stimulus locations. If fixation deviated from the fixation point before the presentation of the target, presentation was prevented immediately and continued to be prevented until accurate fixation occurred again for at least 300 ms. No deviations in fixation occurred during the presentation of each target. Following each target presentation, the screen went blank until a response was made. Participants were required to decide whether the target was a word or pseudoword and to press the appropriate key on the response box. Hand of response was counterbalanced across participants.

### ERP recording and analysis

Continuous electroencephalograph (EEG) signals were recorded by a DC 32-channel amplifier (1-kHz sampling rate, 250 Hz high cut-off frequency; Brain Products Inc., Germany). The EEG activity was recorded via a Waveguard elastic cap, containing 64 unshielded and sintered Ag-AgCl electrodes (CAP-ANTWG64; ANT, Netherlands), with an electrode layout according to the international 10–5 electrode system. The right-earlobe electrode served as on-line reference. EEG waveforms were re-referenced off-line to the average of the right- and the left-earlobe electrodes. Two electrodes placed in a bipolar montage at approximately 1 cm from the outer canthi of both eyes served to record the horizontal electrooculogram (HEOG). The vertical electrooculogram (VEOG) and blinks were recorded from one electrode positioned below the right eye and referenced to the right earlobe. Electrode impedance was kept below 5 KΩ and a notch filter (50 Hz) was used for all recorded channels. EEGs were epoched from 100 ms pre-stimulus-onset to 450 ms post-stimulus-onset. Each EEG epoch was inspected off-line, and those with ocular artefacts (as indicated by HEOG activity exceeding ±40 µV and VEOG activity exceeding ±80 µV) were excluded from statistical analyses.

The latencies of the 3 ERP peaks were measured over the left and right parietooccipital regions (PO7 and PO8) and computed for each target type (word, pseudoword), and target position (LVF Extrafovea, LVF Fovea, RVF Fovea, RVF Extrafovea), relative to a 100 ms pre-stimulus baseline. Parietooccipital regions were chosen because they were likely to reveal effects of early perceptual processes and lexical selection (e.g., [37]–[39]). These ERP recording were also used to reveal the timing of the transfer of information between the two hemispheres (interhemispheric transfer time, IHTT), for the P100, the N170, and the P325. Only ERP data for trials with correct responses were analysed. To help remove slow and sustained shifts in voltage of non-neural origin during data acquisition and reduce high-frequency noise, averaged ERPs were filtered using 1 Hz high-pass and 30 Hz low-pass filters.

## Results

### Behavioural Results

**Figure 1:**
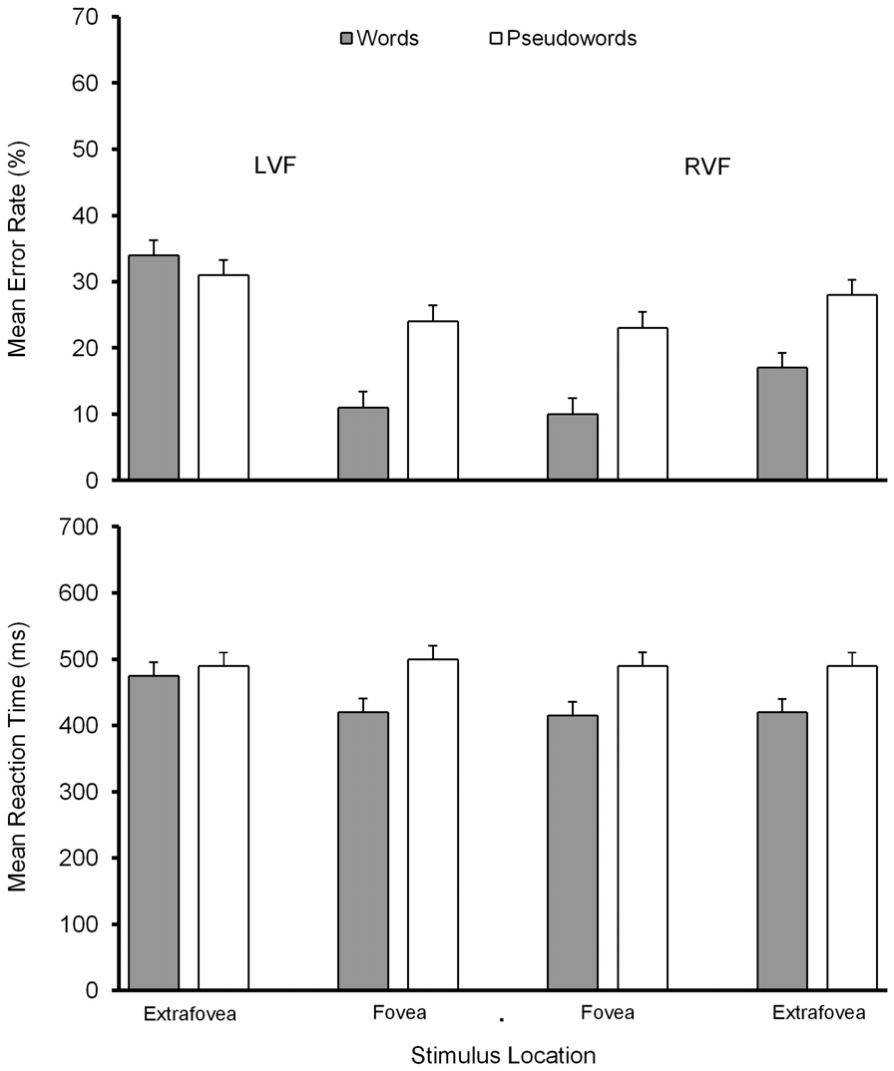
The behavioural results, showing mean error rates and mean reaction times. Bars depict standard errors.

Mean error rates and reaction times for words and pseudowords presented at each foveal and extrafoveal location are shown in Figure 1. ANOVAs with factors lexicality (word, pseudoword), eccentricity (foveal, extrafoveal), and presentation hemifield (left, right) were conducted separately on error rates and on reaction times for accurate responses. Error rates showed main effects of lexicality, *F*(1,11) = 5.97, *p*<0.05, η_p_^2^ = 0.35, eccentricity, *F*(1,11) = 65.56, *p*<0.001, η_p_^2^ = 0.86, and presentation hemifield, *F*(1,11) = 34.08, *p*<0.001, η_p_^2^ = 0.76, and an interaction between all three factors, *F*(3,33) = 8.97, *p*<0.001, η_p_^2^ = 0.45. Extrafoveal locations showed a RVF advantage for words (*p*<0.01) but not pseudowords (*p*>0.05), and a word advantage over pseudowords for RVF presentations (*p*<0.01) but not for LVF presentations (*p*>0.30). Foveal locations showed no hemifield advantage for words or pseudowords (both *p*s>0.05), and similar word-pseudoword advantages for RVF and LVF presentations (*p*s<0.01).

Reaction times showed significant main effects of lexicality *F*(1,11) = 33.70, *p*<0.001, η_p_^2^ = 0.75, and presentation hemifield, *F*(1,11) = 13.45, *p*<0.001, η_p_^2^ = 0.55, but not eccentricity, *F*(1,11) = 3.98, *p*>0.07, η_p_^2^ = 0.27, and an interaction between all three factors, *F*(3,33) = 4.96, *p*<0.01, η_p_^2^ = 0.40. Extrafoveal locations showed a RVF advantage for words (*p*<0.01) but not pseudowords (*p*>0.05), and a word-pseudoword advantage for RVF presentations (*p*<0.01) but not LVF presentations (*p*>0.05). Foveal locations showed no hemifield advantage for words or pseudowords (*p*>0.05), and similar word-pseudoword advantages for RVF and LVF presentations (*p*s<0.01).

### ERP Results

**Figure 2:**
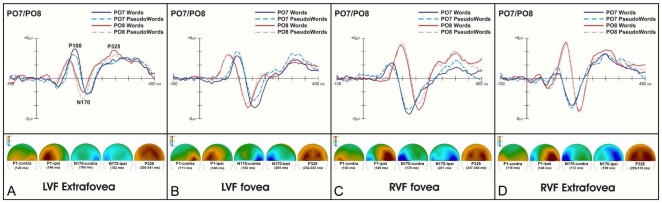
The grand-average ERPs on left/right hemisphere parietooccipital electrodes (PO7/8) and corresponding topographic maps of the P100, N170, and P325 components for words and pseudowords at each of the four stimulus position. The panels A and D show the electrophysiological results for left/right visual field presentations in extrafoveal locations, and panels B and C show the electrophysiological results for left/right visual field presentations in foveal locations.

ERPs and topographic maps of the components analysed over left and right parietooccipital scalp regions are shown in Figure 2. An ANOVA with factors hemisphere (LH, RH), presentation hemifield (LVF, RVF), eccentricity (foveal, extrafoveal), and lexicality (word, pseudoword), was conducted on mean peak latencies for each of the P100, N170, and P325 components.

### P100

Analysis of the P100 showed main effects of hemisphere, *F*(1,11) = 9.40, *p* = .01, η_p_^2^ = .46, due to overall shorter peak latencies for the LH (128 ms) than the RH (133 ms), and eccentricity, *F*(1,11) = 7.43, *p*<.02, η_p_^2^ = .40, due to overall shorter peak latencies for foveal (127 ms) than extrafoveal (133 ms) presentations, and an interaction between hemisphere and presentation hemifield, *F*(1,11) = 205.62, *p*<.001, η_p_^2^ = .95. Post-hoc comparisons showed shorter peak latencies for the hemisphere contralateral (vs. ipsilateral) to the presentation hemifield, for LVF (RH 118 ms vs. LH 146 ms, *p*<.001) and RVF (LH 110 ms vs. RH 148 ms, *p*<.001) presentations. No other effects were significant. Thus, the peak latency of the P100 was significantly delayed over the hemisphere ipsilateral to the hemifield in which stimuli were presented, and this effect did not differ for foveal and extrafoveal locations.

### N170

Analysis of the N170 showed main effects of hemisphere, *F*(1,11) = 34.59, *p*<.001, η_p_^2^ = .76, due to overall shorter peak latencies for the LH (185 ms) than the RH (192 ms), and eccentricity, *F*(1,11) = 10.50, *p*<.05, η_p_^2^ = .49, due to overall longer peak latencies for foveal (193 ms) than extrafoveal (186 ms) presentations, and an interaction between hemisphere and presentation hemifield, *F*(1,11) = 121.62, *p*<.001, η_p_^2^ = 0.92. Post-hoc comparisons showed shorter peak latencies for the hemisphere contralateral (vs. ipsilateral) to the presentation hemifield, for LVF (RH 181 ms vs. LH 199 ms, *p*<.001) and RVF (LH 175 ms vs. RH 203 ms, *p*<.001) presentations. No other effects were significant. Thus, as with the P100, the peak latency of the N170 was significantly delayed over the hemisphere ipsilateral to the hemifield in which stimuli were presented, and this effect did not differ for foveal and extrafoveal locations.

### P325

Analysis of the P325 showed main effects of hemisphere, *F*(1,11) = 22.94, *p*<.001, η_p_^2^ = .67, due to overall longer peak latencies for the LH (335 ms) than the RH (327 ms), presentation hemifield, *F*(1,11) = 24.18, *p*<.001, η_p_^2^ = .69, due to overall longer peak latencies for LVF (337 ms) than RVF (326 ms) presentations, and eccentricity, *F*(1,11) = 230.24, *p*<.001, η_p_^2^ = .95, due to overall longer peak latencies for foveal (350 ms) than extrafoveal (313 ms) displays. There were also two-way interactions of hemisphere and presentation hemifield, *F*(1,11) = 41.40, *p*<.001, η_p_^2^ = .79, hemisphere and eccentricity, *F*(1,11) = 10.33, *p*<.001, η_p_^2^ = .48, presentation hemifield and eccentricity, *F*(1,11) = 5.64, *p*<.05, η_p_^2^ = .34, and presentation hemifield and lexicality, *F*(1,11) = 14.55, *p*<.001, η_p_^2^ = .57. However, these interactive effects were qualified by a three-way interaction of hemisphere, presentation hemifield, and eccentricity, *F*(1,11) = 43.64, *p*<0.001, η_p_^2^ = .80, and a four-way interaction of hemisphere, presentation hemifield, eccentricity and lexicality, *F*(1,11) = 7.79, *p*<.001, η_p_^2^ = .41. Post hoc comparisons for words in extrafoveal locations showed shorter peak latencies for the hemisphere contralateral (vs. ipsilateral) to the presentation hemifield (LVF, RH 297 ms vs. LH 329 ms, *p* = .001; RVF, LH 300 ms vs. RH 311 ms, *p*<.01) but no such effect was observed for foveal locations (LVF, RH 354 ms vs. LH 353 ms, *p* = .76; RVF, LH 352 ms vs. RH 352 ms, *p* = .97). Pseudowords in extrafoveal locations also showed shorter peak latencies for the hemisphere contralateral (vs. ipsilateral) to the presentation hemifield (LVF, RH 302 ms vs. LH 353 ms, *p*<.001; RVF, LH 298 ms vs. RH 309 ms, *p*<.01) but no such effect was observed for foveal locations (LVF, RH 352 ms vs. LH 352 ms, *p* = .87; RVF, LH 345 ms vs. RH 342 ms, *p* = .55).

Finally, RVF presentations in extrafoveal locations produced similar peak latencies for words and pseudowords in each hemisphere (LH, words = 300 ms, pseudowords = 298 ms, *p*>.05; RH, words = 311 ms, pseudowords = 309 ms; *p*>.05). However, LVF presentations in extrafoveal locations produced similar peak latencies for words and pseudowords only in the RH (words = 297 ms, pseudowords = 302 ms, *p*>.05) and a substantial effect of lexicality in the LH (words = 329 ms, pseudowords = 353 ms, *p*<.001). Foveal locations showed similar peak latencies for words and pseudowords in both hemispheres, irrespective of presentation hemifield (all *p*s>.05).

### IHTT

Based on Saron and Davidson [53],we estimated left-to-right and right-to-left IHTT for each of the P100, N170, and P325 by subtracting peak latencies measured over the left and right parietooccipital regions (PO7 and PO8). An ANOVA using the resulting values with factors component (P100, N170, P325), presentation hemifield (LVF, RVF), eccentricity (foveal, extrafoveal), and lexicality (word, pseudoword), showed a significant effect of component, *F*(2,22) = 25.53, *p*<.001, η_p_^2^ = 0.70. No other main effects were significant (*p*>.16). Post-hoc comparisons showed a decrease in overall IHTT from the P100 to the N170 (33 vs. 22 ms, *p*<0.01) and from the N170 to the P325 (22 vs. 13 ms, *p*<0.01). There were also significant two-way interactions of component and presentation hemifield, *F*(2,22) = 35.82, *p*<.001, η_p_^2^ = 0.77, and component and eccentricity, *F*(2,22) = 26.30, *p*<.001, η_p_^2^ = 0.71, and a three-way interaction of these factors, *F*(2,22) = 10.05, *p*<.001, η_p_^2^ = 0.47. Post hoc comparisons revealed faster IHTTs for LVF (vs. RVF) presentations, in both extrafoveal and foveal locations, for the P100 (extrafoveal, 21 ms vs. 32 ms, *p*<.01; foveal, 35 ms vs. 44 ms, *p*<.01) and the NI70 (extrafoveal, 12 ms vs. 27 ms, *p*<.01; foveal, 20 ms vs. 29 ms, *p*<.01). In contrast, IHTTs for the P325 were slower for LVF (vs. RVF) presentations in extrafoveal locations (41 ms vs. 11 ms, *p*<.01) but no difference was observed in foveal locations (1 ms vs. 1 ms, *p*>.40). Finally, there was a significant four-way interaction of component, presentation hemifield, eccentricity, and lexicality, *F*(2,22) = 5.12, *p* = .01, η_p_^2^ = 0.32. Post hoc comparisons showed that lexicality effects were observed only in the P325 and only for extrafoveal displays in the LVF, due to slower IHTTs for pseudowords than for words (50 ms vs. 31 ms, *p*<0.01) at this location. No differences in IHTT were found between words and pseudowords in LVF fovea, RVF fovea, or RVF extrafovea locations (all *p*s>.10).

## Discussion

This research was conducted to investigate the possibility that a division in unilateral projections to each contralateral hemisphere exists in foveal vision, and that this division plays a functional role in foveal word recognition. A paradigm was used in which fixation-contingent displays ensured that words were presented at precisely the retinal locations required in the experiment and so ensured that ERP and behavioural evidence of hemispheric projections and hemispheric processing could be determined accurately for foveal and extrafoveal locations. Words were presented unilaterally to the left or right of fixation at eccentricities that placed them entirely in either foveal or extrafoveal vision, and precautions were taken for foveal presentations to avoid midline areas of bilateral projection and to occupy foveal areas where unilateral contralateral projections are most likely to exist.

The electrophysiological findings show that this approach was successful at revealing an initial division in unilateral projections to each contralateral hemisphere within foveal vision. In particular, when words and pseudowords were presented in each visual hemifield within the fovea, peak latencies produced by the P100 indicated that stimuli were projected first to the hemisphere contralateral to the presentation hemifield, with no indication of concurrent projection to the ipsilateral hemisphere. Similar findings were found for extrafoveal presentations, where unilateral contralateral projections are well established, and this similarity underscores the view that our procedure revealed a genuine division in contralateral hemispheric projections within foveal vision.

However, this finding of an early division in hemispheric projections for both extrafoveal *and* foveal vision contrasts sharply with the behavioural findings which showed clear differences between extrafoveal and foveal locations. In particular, when words were presented in extrafoveal locations, performance (determined both by error rates and reaction times) was superior for presentations in the RVF but the same words presented within the fovea produced very similar levels of performance in each hemifield. Moreover, although the patterns of activity shown by the P100 were unaffected by the lexicality of stimuli (see also [29]), the behavioural findings showed clear effects of lexicality and clear differences between the effects produced by words and pseudowords in extrafoveal and foveal locations. Thus, while the early perceptual nature of the P100 (e.g., [27], [30]–[32], [37]) shows that visual information from extrafoveal locations and from foveal locations away from the midline is initially projected unilaterally to each contralateral hemisphere, this initial division in hemispheric projections appears to lead to a division in extrafoveal word recognition but not to a division in foveal word recognition. Indeed, the latencies produced by the P100 suggest that inter-hemispheric transmission of visual information was well advanced for extrafoveal and foveal locations by 150 ms post stimulus-onset, before even the sub-lexical processing of the N170. In particular, mean IHTT for the P100 was just 33 ms and was fastest from the RH to the LH (28 ms, compared to 38 ms for LH to RH), indicating that both hemispheres soon became activated by stimuli in each hemifield and that information provided by divided projections from both hemifields converged most rapidly on the hemisphere dominant for language.

The N170 continued to show evidence of a division in unilateral projections to each contralateral hemisphere in foveal vision as latencies indicated that stimuli were processed first by the hemisphere contralateral to the presentation hemifield. However, as with the effects observed for the P100, this division too showed no influence of lexicality, suggesting that the division was still sub-lexical. This finding concurs with the widely held view that the N170 reflects the processing of letter identities and letter combinations, which may be applicable generally to orthographically-regular letter strings (e.g., [37], [39], [41]–[43]). Moreover, despite the division in hemispheric projections that was still evident in the N170 for foveal and extrafoveal locations, interhemispheric transmission was also apparent for the N170, suggesting that the interhemispheric transfer of information observed with the P100 continued to produce bilateral sub-lexical letter string processing throughout the early stages of word recognition. Indeed, mean IHTT for the N170 was just 22 ms, compared to 33 ms observed for the P100, indicating that, rather than a persistent and unchanging division in hemispheric processing produced by an initial division in hemispheric projections, activation of both hemispheres was becoming increasingly synchronous. Moreover, as with the P100, this approach towards synchrony was most apparent for transmission from the RH to the LH, where IHTT was just 18 ms (compared to 28 ms for LH to RH), indicating again that both hemispheres soon became activated by stimuli in each hemifield and that information provided by divided projections from both hemifields converged most rapidly on the language-dominant hemisphere.

However, the P325 showed a very different pattern of effects from the P100 and N170. Importantly, the latencies of the P325 revealed differences between words and pseudowords, for foveal and extrafoveal locations, suggesting that word recognition had now occurred. However, these same latencies also revealed that the division in hemispheric processing observed previously was no longer present for foveal locations and remained only for extrafoveal locations. Moreover, these findings closely matched the behavioural findings that foveal locations produced similar performance each side of fixation but extrafoveal locations produced left-right asymmetries. Thus, while extrafoveal locations showed substantial asynchrony in the P325 between hemispheres contralateral and ipsilateral to the hemifield in which stimuli were presented, and clear P325 and behavioural asymmetries in hemispheric processing that were lexically-sensitive, foveal locations showed similar P325 peak latencies in each hemisphere and no indication of any asymmetry (P325 or behavioural) in hemispheric processing.

These findings provide important clues to the nature and function of hemispheric projections in foveal and extrafoveal vision. When using precisely positioned foveal stimuli to avoid midline bilateral projections and exploit foveal areas where unilateral contralateral projections are most likely to exist, an initial division in hemispheric processing was found for extrafoveal and foveal locations. This division appears to exist for extrafoveal and foveal locations at the P100 (approximately 100–150 ms post-stimulus onset), continues in a reduced form for extrafoveal and foveal locations at the N170 (approximately 150–200 ms post stimulus onset) but, by the P325 (approximately 300–350 ms post-stimulus onset), continues for extrafoveal locations only and is completely removed for foveal locations. This suggests that, when recognising a word in foveal vision, the divided projection that occurs initially has already been overcome by inter-hemispheric transmission and no concomitant division exists when word recognition actually takes place. Indeed, our findings suggest that the onset of inter-hemispheric transmission may be boosted for foveal stimuli by the more rapid onset of hemispheric activations (relative to extrafoveal stimuli) observed for the P100 when stimuli were presented in foveal vision. The absence of a functional division for foveal stimuli concurs with views presented by other researchers (e.g., [36]) that, even if human foveae are split anatomically, the transmission of information between the two hemispheres is crucial and may be sufficiently rapid to obviate a functional role for an anatomical divide in foveal word recognition (see also [2], [3], [5], [11], [19]). Indeed, as Dehaene et al. [36] point out, callosal projections beyond V1 may have the structure necessary to ensure the continuity of receptive fields across the foveal midline and allow convergence on common visual representations, which may remove the functional impact of any initial foveal split. The findings of the present study suggest that this may indeed be the case, and that interhemispheric communication within foveal vision produces effective bilateral processing for foveal word recognition.

The clear presence of behavioural evidence, from reaction times and error rates, of a functional division in word recognition for extrafoveal locations indicates that the lexical decision task was well-suited to revealing functional hemispheric asymmetries in word recognition when these occurred and that the absence of evidence of a functional division for foveal locations was not due to the task that was used. Moreover, the overall timing of the P100, N170, and P325 observed in the present study resonates closely with previous estimates of the time course of visual word recognition (e.g., [37], [41]), and their sequence matches indications from fMRI studies of visual word processing [54]. Indeed, given the nature of the lexical decision task and the timing of the P325, our findings suggest that the P325 corresponds to a point when, faced with the requirements of lexical decision, the lexical processor attempts to settle on a single whole-word representation as a unique identification of the stimulus input (i.e., lexical selection; see also [37]). At this point in processing, a whole-word representation may be selected from a number of possible candidates that are compatible with incoming information from the stimulus. In normal reading, this would presumably involve top-down textual influences as well (e.g., [31]) but in the case of the lexical decision paradigm, the process is likely to involve primarily bottom-up input. As processing of the stimulus continues, the array of candidate lexical entries would be refined, and mismatches between input and candidate entries would be used to either select the correct word response or, eventually, to decide that the stimulus is a pseudoword. Such verification may be part of the normal processes involved in accurate word recognition, occurring whenever a mismatch is detected between a selected lexical representation and lower level activation (e.g., [55]). Indeed, this interpretation of the P325 component fits with the findings of other ERP and MEG (magnetoencephalography) studies, where effects thought to reflect lexical identification were found in approximately the same time-window [56]–[58]. Moreover, as Rayner [59] has indicated, findings using the lexical decision task have typically been replicated in normal reading situations where processing is revealed by participants' eye movements [60]–[62].

Crucially, however, it should be noted that the division in foveal projections proposed here and that is supported by the findings of the present research is very different from the division proposed by SFT. In particular, the proposal of SFT (e.g., [63]–[66]) is for a division at the foveal midline that is so precise that each fovea contains only unilateral projections from each hemifield to each contralateral hemisphere with no amount of bilateral overlap, and the division between these contralateral projections produces substantially different effects on foveal word recognition either side of the midline. In contrast, and in line with previous research and numerous concerns and considerations concerning SFT (for reviews, see [3], [11]), it seems more likely that any foveal division lies outside a medial area of bilateral projections and reflects a more graded change in the ratio of bilateral and unilateral projections away from the foveal midline. Moreover, from the findings of the present study, while initial divisions in hemispheric projections in foveal vision away from the midline can be revealed, no evidence of a division that affects foveal word recognition is apparent in the P100, N170, or P325, or in the behavioural findings.

Indeed, while our electrophysiological findings show that processing words in foveal vision involves interhemispheric communication from early-on in processing, the effect of this communication also differs substantially from that proposed by SFT. For example, proponents of SFT argue that foveal information either side of the midline projects separately to each hemisphere but is integrated in the LH (for the majority of individuals who are LH-dominant for language) via interhemispheric transfer prior to lexical processing (e.g., [63]–[65], [67]–[69]). Crucially, however, according to this view, the initial foveal division in hemispheric projections produces a concomitant division in foveal word recognition such that word information to the right of fixation produces a processing advantage because this information projects directly to the superior word recognition capabilities of the LH and does not undergo disruptive interhemispheric transfer prior to LH word recognition. However, while our findings provide evidence of inter-hemispheric communication for foveal stimuli, they provide no evidence that foveal word information is processed better to the right of fixation than to the left, and this concurs with the behavioral findings of other studies, using a range of languages, paradigms, and procedures [24], [25], [33]–[35], [52], [70], [71]. Thus, in contrast to the claims of SFT, the electrophysiological and behavioral findings of the present research extend the findings of previous studies to show that while initial divisions in foveal hemispheric projections may exist, such foveal divisions have no functional relevance for foveal word recognition.

In sum, by using stimuli positioned to avoid midline bilateral projections and to exploit areas where unilateral contralateral projections are most likely to exist, the present study revealed an initial division in hemispheric processing for words in extrafoveal and foveal locations. However, although words in extrafoveal locations produced superior recognition performance in the right visual hemifield, no hemifield division in recognition performance was observed for words in foveal locations. Moreover, while the P100 and N170 latencies indicated that stimuli in foveal and extrafoveal locations were projected first to the hemisphere contralateral to the presentation hemifield, this hemispheric asynchrony decreased substantially for the N170, and the P325 showed a division in hemispheric processing only for extrafoveal locations and no division at all for foveal locations. The indications are, therefore, that even though human foveae can show an initial division in hemispheric projections away from the midline, lexical identification of foveal stimuli involves efficient bilateral processing of foveal input and foveal divisions in hemispheric projections are not functional for foveal word recognition.

## Supporting Information

Footnote S1 (DOCX)

Footnote S2 (DOCX)
