# Supplementary material for: An ERP Assessment of Hemispheric Projections in Foveal and Extrafoveal Word Recognition
Source: PLoS One. 2011 Sep 15;6(9):e23957. doi: 10.1371/journal.pone.0023957 (PMC3174137; doi:10.1371/journal.pone.0023957)
Supplement: Footnote S1 — (DOCX) [file pone.0023957.s001.docx]

Footnote S1. The title of the Gray et al. [1] article contains the phrase “Evidence for unilateral foveal representation” and this has led some researchers (e.g., [2]) to cite this study as support for SFT. In fact, Gray et al. use the term fovea to refer to an area some distance away from the foveal midline, occupying an area between 3 and 5 degrees towards the periphery, and so their findings are not relevant to the notion that a precise split in unilateral foveal projections occurs at the point of fixation and offer no support for SFT.

**References**

1. Gray LG, Galetta SL, Siegal T, Schatz NJ (1997) The central visual field in homonymous hemianopia: Evidence for unilateral foveal representation. Arch Neurol 54: 312-317.

2. Lavidor M, Ellis AW (2003) Interhemispheric integration of letter stimuli presented foveally or extra-foveally. Cortex 39: 69-83.
