# Supplementary material for: An ERP Assessment of Hemispheric Projections in Foveal and Extrafoveal Word Recognition
Source: PLoS One. 2011 Sep 15;6(9):e23957. doi: 10.1371/journal.pone.0023957 (PMC3174137; doi:10.1371/journal.pone.0023957)
Supplement: Footnote S2 — (DOCX) [file pone.0023957.s002.docx]

**Footnote S2.** It was crucial to select a behavioural task that would reveal not just a division in foveal hemispheric projections but also the functionality of such a division for foveal word recognition. Some researchers have used overt naming to asses word recognition (e.g., [1,2]) but speech production is usually lateralised to the LH and so overt naming may produce a spurious advantage for stimuli projected to the LH because this is the hemisphere responsible for producing a response rather than because this is the hemisphere responsible for recognizing those stimuli. In the present study, therefore, naming may have lead to an early division in foveal hemispheric projections being misinterpreted as functional for foveal word recognition. However, the lexical decision task requires lexical access for it to be performed adequately and has been used widely in word recognition and ERP research. Moreover, the task has been shown to be suitably sensitive for revealing hemispheric asymmetries in word recognition and, most importantly, it allows hemispheric confounds in responding to be avoided by appropriately counterbalancing hand of response (e.g., [3]).

**References**

1. Brysbaert M (1994) Interhemispheric transfer and the processing of foveally presented stimuli. Behav Brain Res 64:151-161.

2. Hunter ZR, Brysbaert M, Knecht S (2007) Foveal word reading requires interhemispheric communication. J Cognitive Neurosci 19:1373-1387.

3. Chiarello C, Nuding S, Pollock A (1988) Lexical decision and naming asymmetries: Influence of response selection and response bias. Brain Lang 34: 302-314.
